# Anti-angiogenesis effect of Baiying Juhua Decoction on the non-small cell lung cancer: integrating pharmacology, multi-machine learning and experimental investigation

**DOI:** 10.1186/s40643-025-00993-3

**Published:** 2026-01-24

**Authors:** Xiangwei Meng, Yuan Cao, Qi Shen, Hongyu Zhu, Jianqiao Zhang, Mingxin Dong

**Affiliations:** 1 Department of Medicinal Chemistry, School of Pharmacy, Qingdao University, Qingdao, 266021 China; 2 Department of Drug Clinical Trials, Zibo Central Hospital Affiliated to Binzhou Medical University, Zibo, 255000 China; 3 Central Laboratory, The Affiliated Xuzhou Municipal Hospital of Xuzhou Medical University, Xuzhou, 221000 China; 4 School of Pharmacy, Key Laboratory of Molecular Pharmacology and Drug Evaluation (Yantai University), Ministry of Education, Collaborative Innovation Center of Advanced Drug Delivery System and Biotech Drugs in Universities of Shandong, Yantai University, Yantai, 264005 China

**Keywords:** Non-small cell lung cancer, Network pharmacology, Serum medicinal chemistry, Machine learning, Baiying Juhua Decoction

## Abstract

Baiying Juhua Decoction (BYJHD) is a well-established traditional Chinese herbal formula primarily composed of Solanum lyratum and chrysanthemum, which necessitates a thorough investigation to clarify its mechanisms in combating non-small cell lung cancer (NSCLC). This study employed a combination of network pharmacology predictions, serum pharmacochemistry analysis, and various machine learning algorithms (including LASSO, SVM-RFE, and RF) to identify 38 bioactive compounds that target 653 proteins associated with NSCLC. A cross-analysis of 2161 differentially expressed genes (DEGs) and 3124 functional modules led to the identification of 54 critical therapeutic targets. Following this, protein-protein interaction (PPI) and machine learning analysis pinpointed five key signaling regulators. Molecular docking studies demonstrated strong binding affinities between four representative compounds from BYJHD and these targets. Both in vitro and in vivo experiments confirmed that BYJHD inhibits the progression of NSCLC by exerting anti-angiogenic effects, specifically through the inhibition of the ACVRL-1/Smad/ID-1 signaling pathway and the downregulation of CD34. These findings effectively connect traditional clinical applications with contemporary mechanistic insights, positioning BYJHD as a promising multi-target therapeutic candidate for NSCLC.

**Graphical abstract:**

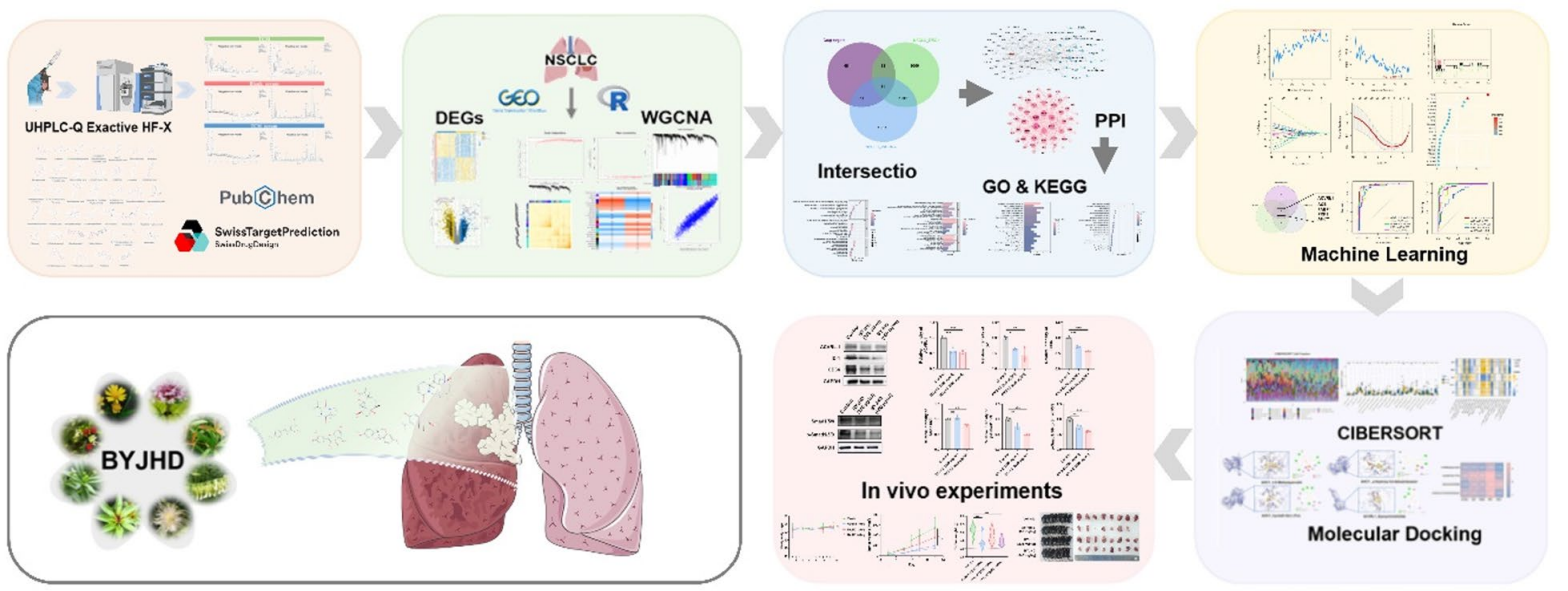

**Supplementary Information:**

The online version contains supplementary material available at 10.1186/s40643-025-00993-3.

## Introduction

Lung cancer remains to be the leading cause of cancer-related morbidity and mortality worldwide, with non-small cell lung cancer (NSCLC) accounting for approximately 85% of all cases (Lahiri et al. 2023; Leiter et al. 2023; Nawaz and Webster 2023; Harada et al. 2023). While molecular targeted therapies, including EGFR and ALK inhibitors, have transformed first-line treatment, their effectiveness in advanced NSCLC is frequently constrained by acquired resistance and tumor heterogeneity (Wang et al. 2023). This therapeutic gap has intensified exploration of complementary strategies, particularly traditional Chinese medicine (TCM), which demonstrates unique advantages in managing symptoms and prolonging survival through its holistic regulatory mechanisms (Fan et al. 2020; Wei et al. 2023; Tian et al. 2024).

TCM’s therapeutic paradigm for NSCLC emphasizes “Fu Zheng Qu Xie” - a dual approach reinforcing host immunity while inhibiting tumor progression (Wei et al. 2023). In contrast to Western pharmacotherapy that often targets single pathways, TCM formulations exert polypharmacological effects through multi-component synergy. Clinically validated formulas such as Bu Fei decoction (Li et al. 2021), Kanglaite injection (Hao et al. 2022), Jinfu’an decoction (Xiong et al. 2014), exemplify this paradigm. Among them, Baiying Juhua Decoction (BYJHD) has shown empirical efficacy in NSCLC through decades of clinical application. Its eight-herb composition (Table 1) composition adheres to the strict “Jun-Chen-Zuo-Shi” formulation principles: Herba Solani Lyrati (Jun herb) coordinates heat clearance and dampness elimination, complemented by Indian Dendranthema (Chen herb) and Herba Clerodendri Bungei (Chen herb) for anti-inflammatory detoxification. Despite its clinical legacy, BYJHD’s anticancer mechanisms remain obscured by phytochemical complexity and insufficient mechanistic validation. To date, no reports have evaluated the antitumor effects and mechanisms of action of BYJHD in non-small cell lung cancer.

Network pharmacology has emerged as a transformative framework for understanding the therapeutic networks of traditional Chinese medicine (TCM), which often involves multiple components and targets (Lu et al. 2023). Grounded in systems biology, this approach aligns with the holistic philosophy of TCM by integrating bioinformatics, cheminformatics, and multi-omics data (Peng et al. 2024). Recent progress in AI-driven target prediction and molecular docking has further improved the mapping of herb-compound-target-pathway interactions (Zhang et al. 2023). Nevertheless, significant challenges remain, particularly concerning database quality and experimental validation of predicted networks (Wang et al. 2022). The rapid advancements in artificial intelligence (AI) and multi-omics sequencing technologies have introduced new opportunities and support for network pharmacology, enabling its application in promoting precision-based traditional Chinese medicine (Zhang et al. 2023; Zhu et al. 2020). These emerging technologies not only enhance the predictive capabilities of network pharmacology but also facilitate a more profound understanding of the complex interplay among herbal compounds, targets, and pathways.

This study initially identified the bioactive components of BYJHD that are absorbed into the bloodstream using metabolomics. Subsequently, network pharmacology and bioinformatics analyses were applied to elucidate the anti-NSCLC mechanisms of these active ingredients, leading to the identification of key targets and the prediction of drug-target interactions through molecular docking and machine learning approaches. The efficacy and underlying molecular mechanisms of BYJHD were further validated through in vitro cellular experiments and in vivo animal models. This systematic investigation provides a robust theoretical basis for the applying BYJHD in NSCLC treatment and presents a new perspective for understanding traditional Chinese medicine mechanisms.

## Materials and methods

### Materials and reagents

The BYJHD contained 30 g of Baiying (*Solanum lyratum Thunb.*), 30 g of YeJuhua (*Chrysanthemum indicum L*), 30 g of Choumudan (*Clerodendrum bungei Steud.*), 15 g of Sankezhen (*Berberis pliretii Schneid.*), 15 g of Kushen (*Sophora flavescens Ait.*), Baitouweng (*Pulsatilla chinensis.*), 15 g of Qiyeyizhihua (*Paris polyphylla Sm.*) and 20 g of Baihuasheshecao (*Scleromitrion diffusum (Willd.) R. J. Wang*). All single herbs were purchased from Zibo Central Hospital Affiliated to Binzhou Medical University (Shandong, China) and authenticated by pharmacy experts. The quality of the herbs met the criteria specified in the 2020 edition of the Chinese Pharmacopoeia. Methanol, formic acid, purified water, ethanol, and acetonitrile were purchased from CNW Technologies GmbH (Düsseldorf, Germany). The antibody used in this study was obtained from Affinity Bioscience (Catalog No. DF3170, DF2932, AF5149, AF0614, AF8313). All chemicals and solvents used were of analytical reagent or chromatographic grade.

## Preparation for BYJHD testing sample

The BYJHD formula, comprising eight herbs (Table 1), was prepared as follows: A total of 170 g of herbs were soaked in ten times their volume of cold distilled water for 45 min and subsequently decocted for 1.2 h. After filtering the solution, the remaining herbal residue was decocted once more in eight times its volume of distilled water for 45 min. The two filtrates were then combined and concentrated to 100 mL using a rotary evaporator (N-1300, Shanghai Ailang Instrument Co., Ltd., Shanghai, China). The final concentration of the BYJHD decoction was 1.7 g (crude drug)/mL, and the solution was stored at −20 °C for subsequent use.

**Table 1:** Herbal composition and dosage of BYJHD

Chinese name	English name	Scientific name	Weight (g)
Baiying	Herba Solani Lyrati	*Solanum lyratum Thunb.*	30
Yejuhua	Flower of Indian dendranthema	*Chrysanthemum indicum L.*	30
Choumudan	Herba clerodendri bungei	*Clerodendrum bungei Steud.*	30
Sankezhen	Barberry	*Berberis pliretii Schneid.*	15
Kushen	Sophorae flavescentis radix	*Sophora flavescens Ait.*	15
Baitouweng	Pulsatillae radix	*Pulsatilla chinensis (Bge.)*	15
Qiyeyizhihua	Polyphylla Paris rhizome	*Paris polyphylla Sm.*	15
Baihuasheshecao	Spreading hedyotis herb	*Scleromitrion diffusum (Willd.) R. J. Wang*	20

## Animal experiments

All animal experiments were conducted in compliance with institutional animal care guidelines and approved protocols (approval No. ZXYY202207006). A total of 32 male C57BL/6J mice (8 weeks old) were used in the study. To establish the xenograft tumor model, 5 × 10⁶ LLC cells (Lewis lung carcinoma) mixed with Matrigel (Beyotime, China) were subcutaneously injected into the right flank of each mouse. The mice were randomly assigned to four groups (8 mice per group) based on body weight. The treatment groups and corresponding drug regimens were as follows: negative control group, administered CMC-Na intragastrically every day; positive control group, administrated gefitinib (50 mg/kg) intragastrically; and treatment group, administered BYJHD intragastrically at doses of 320 mg/kg and 640 mg/kg every day. Drug treatment was initiated when tumor volumes reached 20 ± 7 mm³ (mean ± SD). Tumor volume and body weight were recorded regularly. Tumor volume was calculated using the following formula: tumor volume (mm^3^) = π/6 × (length) × (width)^2^. After 13 days of treatment, all mice were euthanized. Tumors were excised, weighed, and recorded for further analysis.

## Preparation of BYJHD-containing serum

After a 7-day acclimatization period, 20 rats were randoml y assigned to either the serum-containing group or the control group. The serum-containing group was further divided into low-dose, medium-dose, and high-dose BYJHD subgroups, with 3 rats in each (*n* = 3 per subgroup). Rats in the serum-containing subgroups were administered BYJHD via gavage twice daily for 5 consecutive days at a dosage of 0.2 mL/100 g per administration. The control group, consisting of 11 rats, received saline by gavage.

One hour following the final administration on Day 5, the rats were anesthetized with pentobarbital sodium. Blood samples were then drawn from the abdominal aorta. Serum was isolated through centrifugation at 4 °C, spinning at 3500 rpm for 15 min, and subsequently subjected to heat inactivation in a water bath set at 56 °C for 30 min. The serum was filtered using a 0.22 μm microporous membrane to remove contaminants and subsequently stored at −20 °C for future use.

## Sample preparation for the metabolomics experiment

For untargeted metabolomics analysis, serum samples were thawed at 4 °C and vortexed for 5 s at room temperature. Subsequently, 0.5 mL of serum was combined with 0.5 mL of methanol/acetonitrile (v/v = 1:1) in a 2 mL tube and vortexed for 60 s. The mixture was then centrifuged at 12,000 rpm for 20 min at 4 °C. The supernatant was stored at −80 °C until UPLC-QTOF/MS analysis. Furthermore, 100 µL aliquots of the supernatant were pooled to create quality control (QC) samples to ensure reproducibility.

### UHPLC-Q exactive HF-X analysis

The composition of the serum containing BYJHD was qualitatively analyzed using UHPLC-Q Exactive HF-X. Liquid chromatography separation was conducted using a Waters HSS T3 column (100 × 2.1 mm, 1.8 μm) at a column temperature of 40 °C, with a flow rate of 0.3 L/min, and an injection volume of 2 µL. The mobile phase consisted of 0.1% formic acid in water (Phase A) and 0.1% formic acid in a mixture of acetonitrile and isopropyl alcohol (Phase B). The gradient elution program was as follows: 0–2 min A/B (90:10, v/v), 2–6 min A/B (90:10, v/v), 6–15 min (VA/VB = 40:60), and 15.1–17 min (H_2_O: acetonitrile = 90:10).

The Q Exactive HFX high-resolution mass spectrometry was utilized to acquire first and second-order spectra. The ESI parameters were as follows: sheath gas at 40 psi; auxiliary gas at 10 psi; ion spray voltage at 3000 V for positive mode and − 2800 V for negative mode; temperature at 350 °C; ion transfer tube temperature at 320 °C. The scan mode was Full-scan MS2; the scanning method was in both positive and negative ion modes. The first-order scan range (scan m/z range) was 70-1050 Da, with a first-order resolution of 70,000 and a second-order resolution of 17,500.

QC samples were injected every six samples to ensure experimental stability. Raw MS data were processed using Progenesis QI software (Waters Corporation, Milford, USA) for peak alignment, peak picking, and annotation. Peaks were identified using a self-built secondary MS database for Chinese herbal medicine, applying corresponding fragmentation rule-matching methods. All UHPLC-Q Exactive HF-X operations and data analyses were performed in collaboration with the Zibo Central Hospital Drug Clinical Trials Center.

## Network pharmacology-based analysis

The 2D and 3D structures of all compounds in canonical SMILES and SDF formats were obtained from the PubChem database (https://pubchem.ncbi.nlm.nih.gov/). The Swiss Target Prediction database (http://www.swisstargetprediction.ch/) was utilized to retrieve the putative targets of chemical components identified by UHPLC-Q Exactive HF-X analysis. Targets were aligned to gene names via the UniProt database (www.uniprot.org/). Chemical compounds that could not be associated with relevant targets were excluded from further analysis. Finally, the Target information from the Swiss Target Prediction database was summarized and refined to obtain the drug target of BYJHD in this study.

## Prediction of disease targets for lung cancer and weighted gene co-expression network analysis (WGCNA) analysis

NSCLC-related target genes were identified by searching the Gene Expression Omnibus (GEO) database using the keyword “non-small cell lung cancer.” Data from the GSE19804 dataset, based on the GPL570 [HG-U133_Plus_2] Affymetrix Human Genome U133 Plus 2.0 Array platform, were downloaded for analysis. The R package Limma was utilized to identify differentially expressed genes (DEGs) between tumor and adjacent normal NSCLC tissues, employing a cutoff of |log2FC| > 1 and a *p*-value < 0.05. Subsequently, WGCNA was applied to systematically analyze DEGs based on weighted correlations and to construct gene co-expression networks.

### Establishment of the protein-protein interaction (PPI) network

The intersected target genes were uploaded to the STRING database (http://string-db.org/cgi/input.pl) to obtain protein-protein interaction data. Cytoscape v3.7.2 was then utilized to visualize and analyze the PPI network, revealing the correlations and relationships between drug targets and active ingredients.

### Enrichment analysis of the potential target genes

Functional enrichment analysis was conducted on potential target genes to investigate their biological roles and associated signaling pathways. The analyses were performed using the Gene Ontology (GO) and Kyoto Encyclopedia of Genes and Genomes (KEGG) pathways, with the aid of the clusterProfiler R package.

### Identifying hub genes

LASSO logistic regression analysis was conducted using the glmnet R package, with the optimal minimal lambda value determined via 10-fold cross-validation to ensure that the partial likelihood deviation met the minimum criteria. Support vector machine-recursive feature elimination (SVM-RFE) analysis was performed using the e1071 and svmRadial R packages with fivefold cross-validation, while the Random Forest (RF) algorithm in the randomForest R package was utilized to analyze the intersecting genes. Hub genes were identified by intersecting the results from the three machine learning methods (LASSO, SVM-RFE, and RF) using a Venn diagram. Finally, ROC curve analysis was performed on the test dataset GSE19804 and validation dataset GSE10072 to validate the diagnostic value of the model.

### Immune infiltration and immune checkpoint-related genes analysis

CIBERSORT was utilized to estimate the relative proportions of 22 infiltrating immune cell types (http://cibersortx.stanford.edu). The Spearman rank correlation test, executed in R, was utilized to evaluate the associations between infiltrating immune cells and between hub genes and immune cell proportions. The correlation results were visualized using the ggplot2 R package. The immune infiltration analysis was performed using the GSE19804 dataset from GEO, combined with a reference immune cell gene expression dataset. The immune cell fingerprint was generated based on immune-related gene expression patterns. Spearman correlation analysis was conducted to identify the strongest associations between hub genes and immune cell types.

### Molecular docking

To investigate the interactions between active compounds and key targets, molecular docking analysis was conducted. The mol2 files of four primary active ingredients were retrieved from PubChem, and the crystal structures of the core target proteins were sourced from the Protein Data Bank (PDB, https://www.rcsb.org/). The molecular docking studies were executed using AutoDockVina version 1.1.2. The binding energy, less than − 5.0 kcal/mol, served as the criterion for evaluating the reliability of predictions, with ligand-macromolecule complexes exhibiting lower binding energy being considered more favorable structures. Finally, the Pymol program was utilized to visualize the binding patterns.

### Cell viability assay

The LLC cells were obtained from the American Type Culture Collection (ATCC, Manassas, VA, USA), and the A549 cells were kindly provided by Professor Hongbo Wang of the School of Pharmacy, Yantai University. Both cell lines were cultured in DMEM medium supplemented with 10% fetal bovine serum (FBS), 100 U/mL penicillin, and 100 mg/mL streptomycin. Cells were maintained at 37°C in a humidified incubator with 5% CO_2_. A549 and LLC cells (5,000 cells/well) were seeded in 96-well plates and allowed to adhere overnight. The cells were then treated with varying concentrations of medicated serum containing Compound BYJHD for 48 h. Following treatment, 10 µL of the enhanced CCK-8 solution (E-CK-A362, Elabscience, China) was added to each well, and the plates were incubated at 37°C for an additional 4 h. The absorbance at 450 nm was measured using a microplate reader (Molecular Devices, San Jose, CA, USA).

### Quantitative real‑time polymerase chain reaction (qPCR)

Total RNA was extracted from A549 cells using Trizol reagent (Invitrogen, Carlsbad, CA, USA). Complementary DNA (cDNA) was synthesized using the reverse transcription kit (Sparkjade, China) following the manufacturer’s instructions. The synthesized cDNA was amplified with specific primers (Sparkjade, China). The qPCR parameters were set as follows: denaturation at 95 °C for 3 min; 40 cycles of 95 °C for 15 s (denaturation); and 60 °C for 31 s (annealing). Data were analyzed using the 2^−ΔΔCT^ method. Primer sequences are listed in Table 2.

**Table 2:** Sequences of quantitative real-time PCR primers

Gene name	Primer	Sequence (5’→3’)
ACVRL1	Forward	CGAGGGATGAACAGTCCTGG
	Reverse	GTCATGTCTGAGGCGATGAAG
ACE	Forward	CGAAGCCGAAGACCTGTTCTA
	Reverse	GGGCAAGTGTGGACTGTTCC
FABP4	Forward	ACTGGGCCAGGAATTTGACG
	Reverse	CTCGTGGAAGTGACGCCTT
PRF1	Forward	GGCTGGACGTGACTCCTAAG
	Reverse	CTGGGTGGAGGCGTTGAAG
AHCY	Forward	ATTCCGGTGTATGCCTGGAAG
	Reverse	GAGATGCCTCGGATGCCTG
ACTIN	Forward	GAAGATCAAGATCATTGCTCCT
	Reverse	TACTCCTGCTTGCTGATCCA

### Wound-healing assay

A549 cells were incubated in 6-well plates until they reached 100% confluence. A denuded area was created by scrapping with a 200 µL plastic pipette tip on the cell monolayer. The medium was then removed, and the monolayer was washed three times with PBS. The cells were subsequently starved for 24 h by incubating them in medium lacking FBS. Following this, medium containing varying concentrations of medicated serum containing Compound BYJHD (100 and 200 µg/mL) or gefitinib (1 µM) was added to each well. Cell movements into the wound area were observed after 0, 24, and 72 h of incubation using a microscope. The recovered wound area was analyzed using ImageJ software.

### Western blotting

Tumor tissue was lysed with RIPA buffer containing protease inhibitors (1% PMSF, 0.5% aprotinin, 0.5% leupeptin) and phosphatase inhibitors (1 mM Na_3_VO_4_, 1 mM NaF) on ice for 30 min. The lysates were centrifuged at 14,000 rpm for 10 min at 4 °C, and the protein concentration was determined using a bovine serum albumin (BSA) standard (Beyotime, China). Equal amounts of protein from each sample were resolved by sodium dodecyl sulfate-polyacrylamide gel electrophoresis (SDS-PAGE) and transferred onto polyvinylidene fluoride (PVDF) membranes (Biorad, USA). The membranes were blocked with 5% nonfat milk in Tris-buffered saline with Tween 20 (TBST; 10 mmol/L Tris, 150 mmol/L NaCl, 1% Tween 20, pH 7.4) for 1 h at room temperature. The blots were then incubated with primary antibodies (anti-ACVRL1, anti-Smad1/5/9, anti-P-Smad1/5/9, anti-ID-1, and anti-CD34 antibody at 1:1000; Affinity, China) at 4 °C overnight. After washed with TBST buffer for three times, the blots were incubated with the secondary antibody (Beyotime, China) for 2 h at room temperature. Immunoreactivity was determined using an advanced ECL kit (Thermofisher, USA) and visualized using a chemiluminescence imaging system (Biorad).

### Statistical analysis

The experimental data were analyzed by GraphPad Prism 9.0 software with a t-test for comparison between two groups and a one-way ANOVA for comparison among multiple groups, and Bonferroni’s test employed for post hoc analysis.

## Results

### Metabolomics analysis of absorbable BYJHD components

**Fig. 1:**
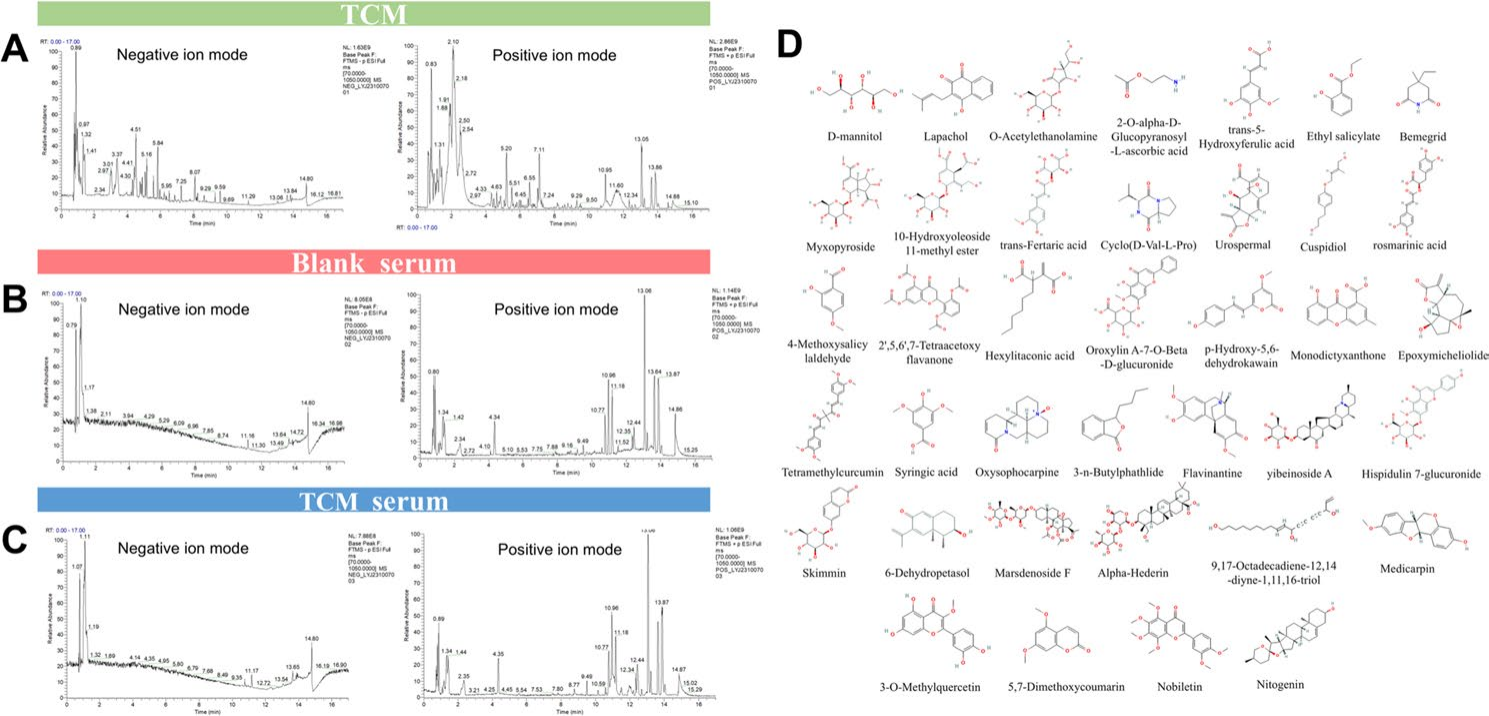
The total ion chromatograms of different BYJHD samples by UHPLC-Q Exactive HFX. **A** BYJHD aqueous extract; **B** Rat serum; **C** BYJHD-treated rat serum; **D** the 38 blood-absorbed active components of BYJHD

An UHPLC-Q Exactive HFX method was developed to identify the blood-entering compounds in the BYJHD formula. Candidate compounds from the medicated serum samples were extracted from positive and negative ion chromatograms using secondary fragment ion information and partial quality control spectra (Fig. 1A-C). To ensure specificity, peaks with matching masses and fragment intensities found in blank serum were excluded. After subtracting endogenous components detected in blank plasma, 38 blood-absorbed compounds were identified, comprising 22 in negative ion mode and 16 in positive ion mode. Detailed information for each compound is provided in Tables 3 and their structures are shown in Fig. 1D.

**Table 3:** Identification of the blood-entering phytochemical components of the BYJHD

NO.	Compound Identification	Rt (min)	Formula	Adducts	Measured value (m/z)
01	D-mannitol	0.766	C_6_H_14_O_6_	[M-H]^−^	181.072
02	Lapachol	0.894	C_15_H_20_O_9_	[M + FA-H]^−^	389.110
03	2-O-alpha-D-Glucopyranosyl-L-ascorbic acid	0.875	C_12_H_18_O_11_	[M-H]^−^	337.078
04	O-Acetylethanolamine	1.087	C_4_H_9_NO_2_	[M-H]^−^	102.056
05	trans-5-Hydroxyferulic acid	3.041	C_10_H_10_O_5_	[M + FA-H]^−^	255.051
06	Ethyl salicylate	3.846	C_9_H_10_O_3_	[M + FA-H]^−^	211.061
07	Bemegrid	3.989	C_8_H_13_NO_2_	[M + FA-H]^−^	200.093
08	Myxopyroside	4.094	C_18_H_26_O_13_	[M-H]^−^	449.131
09	10-Hydroxyoleoside-11-methyl ester	4.480	C_17_H_24_O_12_	[M-H]^−^	419.120
10	trans-Fertaric acid	4.986	C_14_H_14_O_9_	[M-H]^−^	325.057
11	Cyclo(D-Val-L-Pro)	5.305	C_10_H_16_N_2_O_2_	[M + FA-H]^−^	241.120
12	Urospermal	5.939	C_15_H_18_O_5_	[M-H]^−^	277.109
13	Cuspidiol	6.092	C_14_H_20_O_3_	[M + FA-H]^−^	281.140
14	rosmarinic acid	6.391	C_18_H_16_O_8_	[M-H]^−^	359.078
15	4-Methoxysalicylaldehyde	6.391	C_8_H_8_O_3_	[M-H_2_O-H]^−^	133.030
16	2’,5,6’,7-Tetraacetoxyflavanone	6.932	C_23_H_20_O_10_	[M + FA-H]^−^	501.104
17	Hexylitaconic acid	7.157	C_11_H_18_O_4_	[M-H]^−^	213.113
18	Oroxylin A-7-O-Beta-D-glucuronide	7.157	C_22_H_20_O_11_	[M-H]^−^	459.094
19	*p*-Hydroxy-5,6-dehydrokawain	7.237	C_14_H_12_O_4_	[M + FA-H]^−^	289.072
20	Monodictyxanthone	8.774	C_15_H_10_O_5_	[M-H_2_O-H]^−^	251.035
21	Epoxymicheliolide	9.209	C_15_H_20_O_4_	[M-H_2_O-H]^−^	245.119
22	Tetramethylcurcumin	10.137	C_25_H_28_O_6_	[M-H]^−^	423.182
23	Syringic acid	4.099	C_9_H_10_O_5_	[M + H]^+^	199.060
24	Oxysophocarpine	4.378	C_15_H_22_N_2_O_2_	[M + H]^+^	263.175
25	3-n-Butylphathlide	5.071	C_12_H_14_O_2_	[M + H-H_2_O]^+^	173.096
26	Flavinantine	5.700	C_19_H_21_NO_4_	[M + H-H_2_O]^+^	310.143
27	yibeinoside A	5.807	C_33_H_53_NO_7_	[M + H]^+^	576.389
28	Hispidulin 7-glucuronide	6.315	C_22_H_20_O_12_	[M + H]^+^	477.102
29	Skimmin	6.341	C_15_H_16_O_8_	[M + H]^+^	325.091
30	6-Dehydropetasol	6.620	C_15_H_20_O_2_	[M + H-H_2_O]^+^	215.143
31	Marsdenoside F	6.800	C_39_H_60_O_14_	[M + H]^+^	753.405
32	Alpha-Hederin	6.971	C_41_H_66_O_12_	[M + H]^+^	751.462
33	9,17-Octadecadiene-12,14-diyne-1,11,16-triol	7.194	C_18_H_26_O_3_	[M + NH_4_]^+^	308.221
34	Medicarpin	7.272	C_16_H_14_O_4_	[M + H]^+^	271.096
35	3-O-Methylquercetin	7.312	C_16_H_12_O_7_	[M + H]^+^	317.065
36	Citropten	8.940	C_11_H_10_O_4_	[M + H]^+^	207.065
37	Nobiletin	9.355	C_21_H_22_O_8_	[M + H]^+^	403.138
38	Nitogenin	10.294	C_27_H_42_O_3_	[M + H]^+^	415.320

### BYJHD drug potential targets and NSCLC disease targets acquisition

**Fig. 2:**
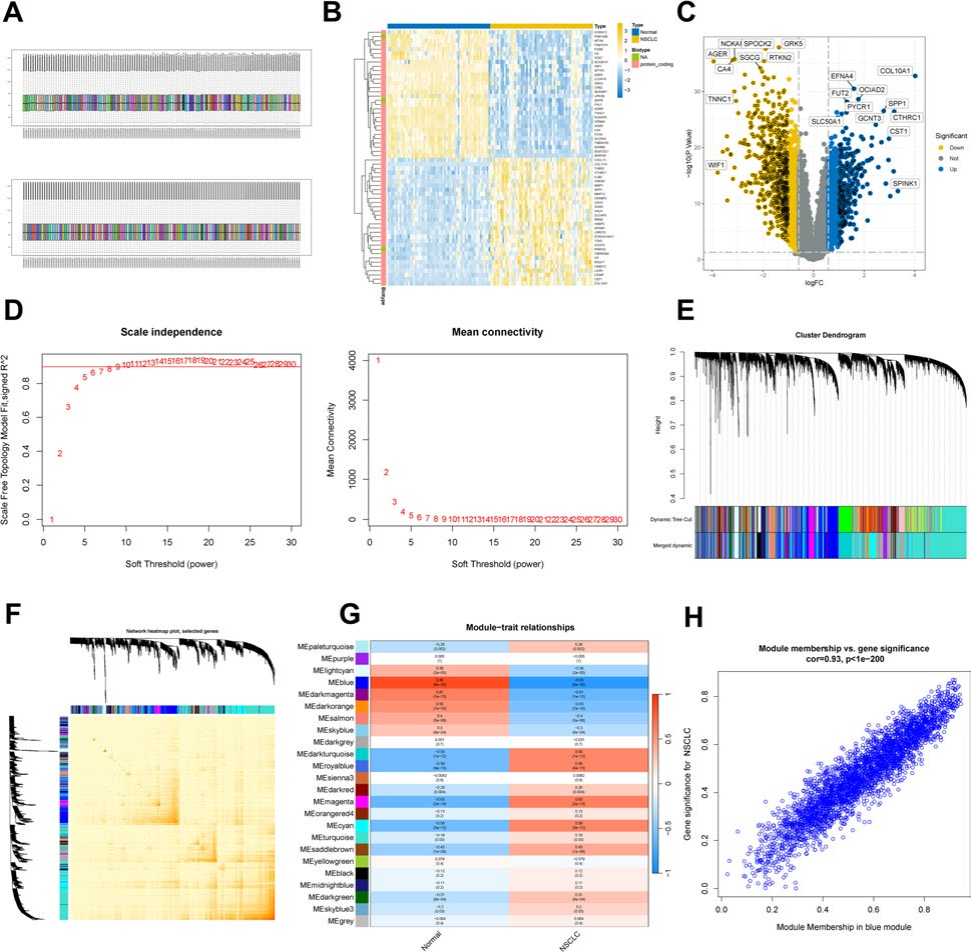
Identification of NSCLC disease targets from the GSE19804 Dataset. **A** Dataset before and after normalization, **B** the heatmap of the top fifty DEGs, with blue representing the normal group and yellow representing the NSCLC group, **C** the volcano plot of DEGs, blue and yellow circles indicate up- and down- regulated DEGs. **D**–**H** identification of related modules associated with NSCLC by weighted gene co- expression network analysis (WGCNA), **D** β = 6 was regarded the optimal soft- power value according to scale independence and average connectivity, **E** a gene clustering tree comprising multiple divided modules, with different clusters appended in varying hues, **F** topological Overlap Matrix (TOM) Heatmap, lighter colors indicate higher overlap, **G** the correlation between each co-expression module and clinical traits. Blue and red represent negative and positive correlations, respectively, **H** the relationship between gene significance for NSCLC and module membership for genes in the blue module

The Swiss Target Prediction database was utilized to identify 653 potential targets corresponding to the 37 active ingredients in the BYJHD formula. To ensure consistency and accuracy in the analysis, batch effects between samples in the NSCLC dataset were removed, and the dataset was normalized (Fig. 2A). Differential expression analysis of the normalized dataset performed with the limma R package, revealed 2,161 differentially expressed genes (DEGs). These DEGs consisted of 871 upregulated genes and 1,290 downregulated genes (Fig. 2B and C).

Weighted Gene Co-Expression Network Analysis (WGCNA) was performed to construct co-expression networks and identify gene modules associated with NSCLC and normal individuals. The variance of gene expression for each gene in the GSE19804 dataset was calculated, and the top 25% of genes with the highest variance were selected for further analysis. A soft-threshold power of 6 was chosen to identify co-expressed gene modules, which ensured a scale-free R² value reached 0.9 (Fig. 2D). Using the dynamic tree cut algorithm, 24 distinct co-expression modules were obtained, each represented by a different color, alongside a Topological Overlap Matrix heatmap (TOM) (Fig. 2E and F). Next, the relationship between the identified modules and clinical features was analyzed. The blue module demonstrated the strongest association with NSCLC, comprising a total of 3,124 genes (Fig. 2G). Further analysis revealed a negative correlation between the genes in the blue module and various NSCLC samples, suggesting a potential role in disease progression (Fig. 2H).

### Target network construction of BYJHD active compounds for treating NSCLC and protein-protein interaction (PPI) analysis

**Fig. 3:**
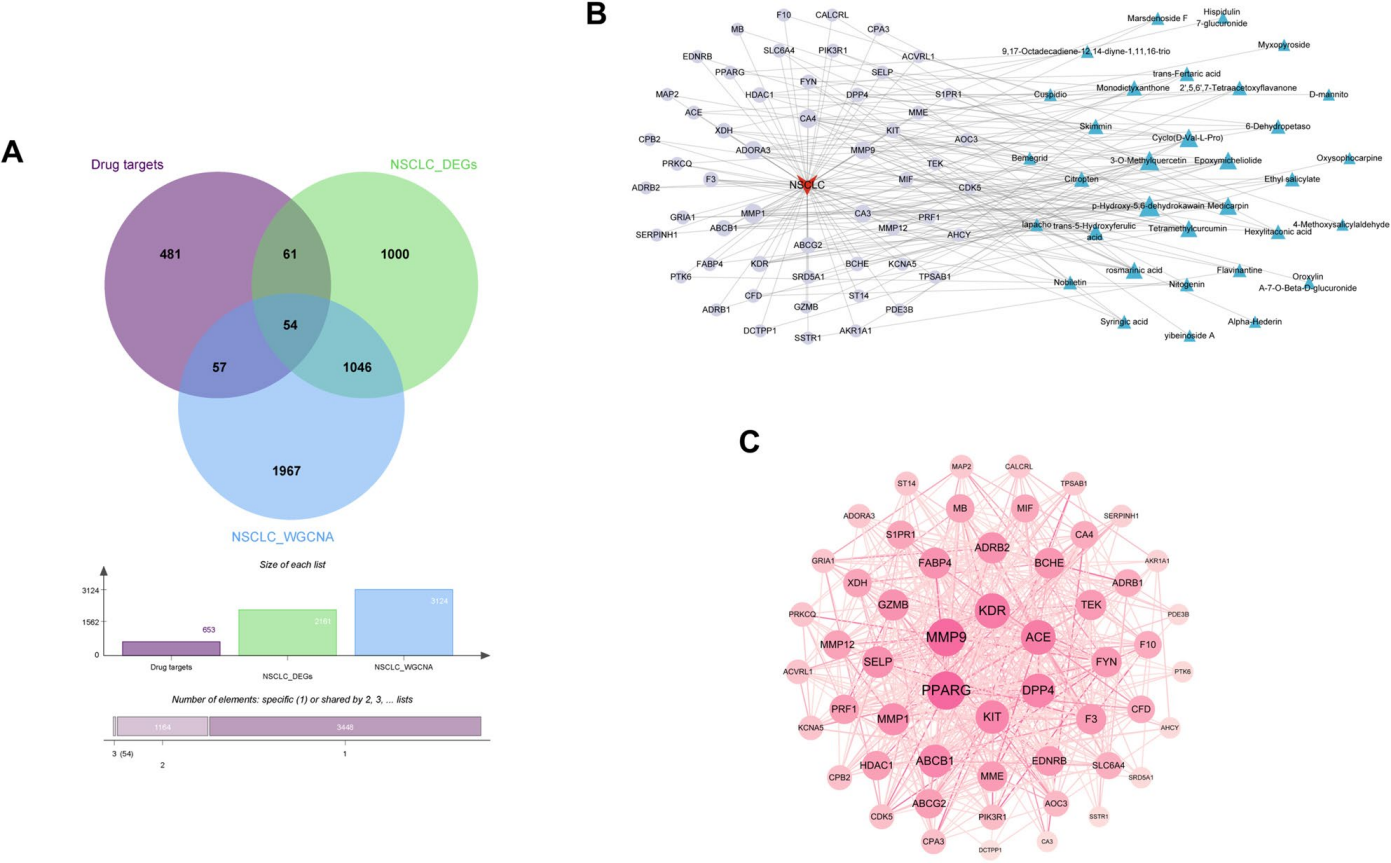
Construction and evaluation of potential therapeutic target networks. **A** Venn diagram of BYJHD active compound targets, NSCLC differential genes, and WGCNA-associated genes, **B** the interactive network of the active compounds and related target genes. Triangles represent active compounds, and circles represent the key targets of drug active compounds corresponding to the disease. The edges indicate interaction relationships between the nodes, **C** the PPI network of genes for BYJHD treatment of NSCLC, deeper colors and larger node sizes indicate higher degree values

As shown in Fig. 3A and B, 54 intersecting genes were identified by overlapping the BYJHD drug targets with NSCLC-related differential genes and WGCNA-associated targets. The resulting “active ingredient - disease target” network, constructed in Cytoscape, consisted of 88 nodes (33 active compounds, 54 targets, and 1 disease) and 186 edges. Topological analysis yielded an average of 4.227 adjacent nodes, a heterogeneity of 1.386, density of 0.049, and centrality of 0.585, highlighting a multi-component, multi-target regulatory mechanism. Core nodes such as p-Hydroxy-5,6-dehydrokawain (Degree = 12), 3-O-Methylquercetin (Degree = 10), Cyclo(D-Val-L-Pro) (Degree = 9) and Epoxymicheliolide (Degree = 8) (Table S1), emerged as central hubs with strong therapeutic potential for NSCLC.

Subsequently, the 54 intersecting targets identified were imported into the STRING database to construct a protein-protein interaction (PPI) network comprising 54 proteins and 537 interaction edges. The network was visualized and analyzed, as shown in Fig. 3. Based on degree centrality, the top 20 targets were identified as core targets (Table S2), which are believed to play pivotal roles in the pathogenesis and progression of non-small cell lung cancer (NSCLC) treated with BYJHD.

### GO enrichment analysis and KEGG signaling pathway analysis

**Fig. 4:**
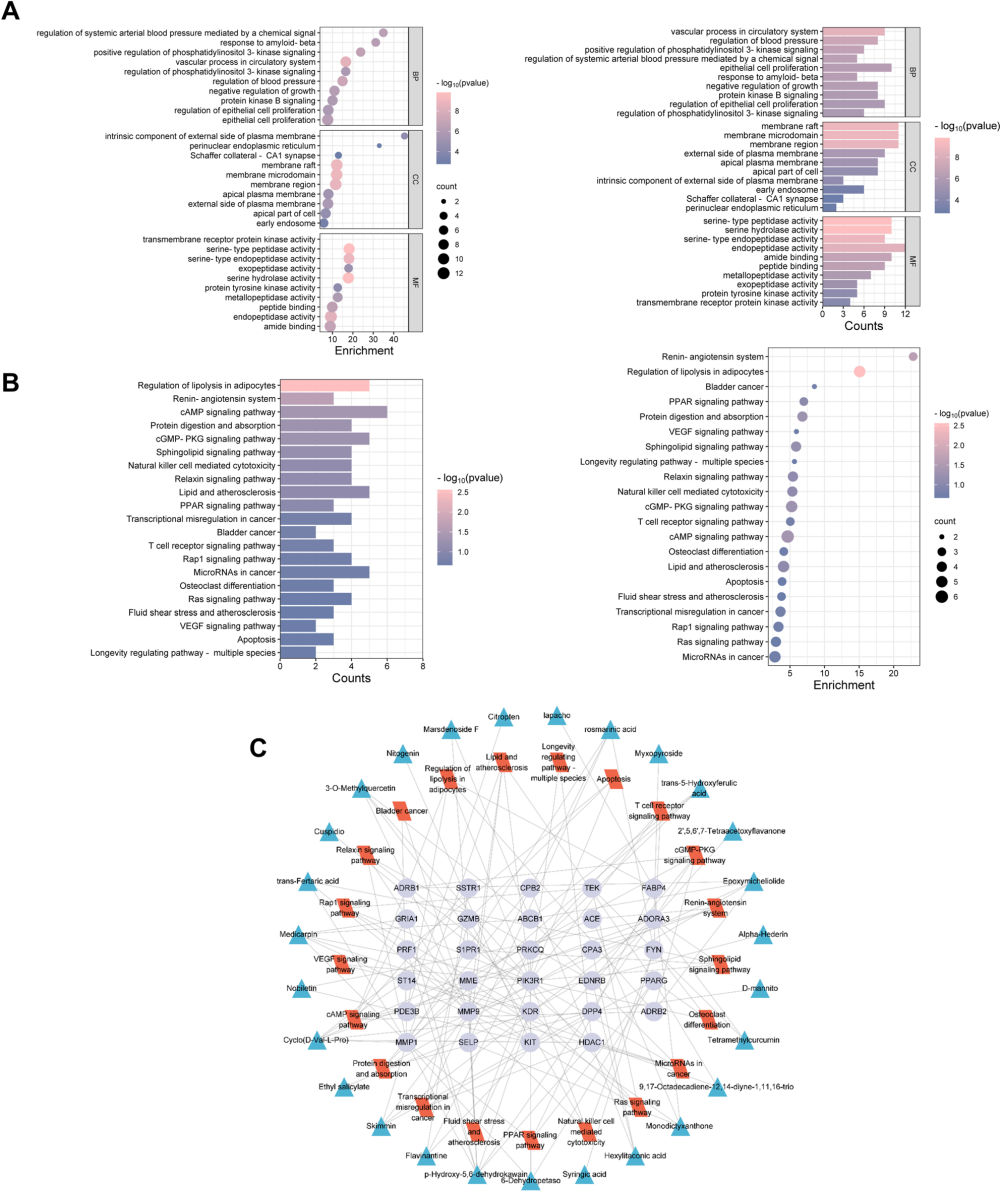
Analyses of the potential GO terms and KEGG signaling pathways of BYJHD for treatment of NSCLC. **A** The top 10 biological process (BP), molecular function (MF) and cellular component (CC) in GO analysis, **B** representative pathways in the KEGG signaling pathway analysis of intersecting genes, sorted by *p*-value, **C** visualization of active ingredient-target-linkage network diagrams

Further analysis of the 54 intersecting targets was conducted to investigate their gene functions, including Gene Ontology (GO) enrichment analysis, which identified a total of 1,449 terms. The biological processes primarily involved included vascular process in circulatory system, positive regulation of phosphatidylinositol 3-kinase signaling, and epithelial cell proliferation. Regarding molecular functions, the targets were mainly associated with the regulation of enzymatic activities, such as transmembrane receptor protein kinase, serine peptidase, and protein tyrosine kinase activities. The cellular components encompassed membrane raft, membrane microdomain, membrane region, and external side of plasma membrane (Fig. 4A).

Additionally, KEGG pathway enrichment analysis revealed 35 pathways (q-value < 0.05) associated with the intervention of BYJHD in NSCLC (Fig. 4B). Key pathways closely related to cancer initiation and progression included transcriptional dysregulation in cancer, Rap1 signaling pathways, Ras signaling pathways, and cAMP signaling pathways. Metabolic pathways such as cGMP-PKG signaling pathways, renin-angiotensin system was implicated in the regulation of the complex tumor microenvironment. The constructed ‘active component-target-pathway’ network demonstrated the connections between active components, targets, and multiple signaling pathways (Fig. 4C).

### Screening of candidate targets via multi-machine learning methods

**Fig. 5:**
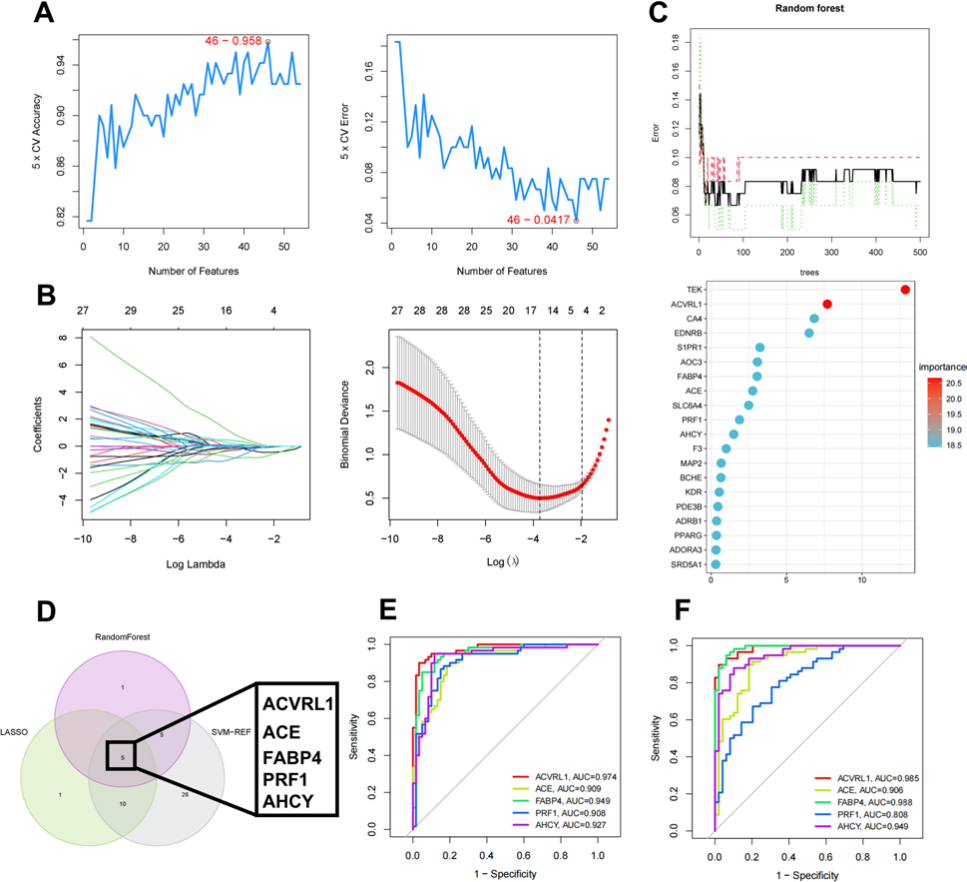
Selection candidate core targets associated with NSCLC progression via multiple machine learning algorithms. **A** 46 significant feature genes were selected from a set of 54 intersecting genes via SVM-RFE algorithm. Maximal accuracy = 0.958, RMSE = 0.0417. **B** in the LASSO regression model, the important feature genes corresponding to the lowest point of the curve based on 10-fold cross-validation were identified as 16 genes, **C** 11 characterized target genes were selected based on relative importance using the random forest algorithm, **D** the Venn diagram illustrating the intersection of screening results from three machine learning algorithms, **E** ROC Analysis of test dataset GSE19804, **F** ROC analysis of validation dataset GSE10072

In the core genes, candidate targets for further research were identified using three machine learning algorithms (LASSO, SVM-RFE, and RF). In the SVM-RFE algorithm, the top 46 genes among the 54 intersecting targets exhibited the smallest prediction error and the highest accuracy in NSCLC prediction (Fig. 5A). In the LASSO algorithm, 16 genes displayed the minimal binomial deviance on the curve (Fig. 5B). Additionally, the RF algorithm selected 11 genes with an importance value greater than 1 (Fig. 5C). Finally, by integrating the results from all three machine learning algorithms, five candidate targets were identified: ACVRL1, ACE, FABP4, PRF1, and AHCY (Fig. 5D). The identified core target genes demonstrated an area under the ROC curve exceeding 0.8 in both the training and validation datasets, indicating the machine learning model’s accurate discrimination capability between lung cancer samples and normal samples (Fig. 5E and F).

### Analysis of immune cell infiltration

**Fig. 6:**
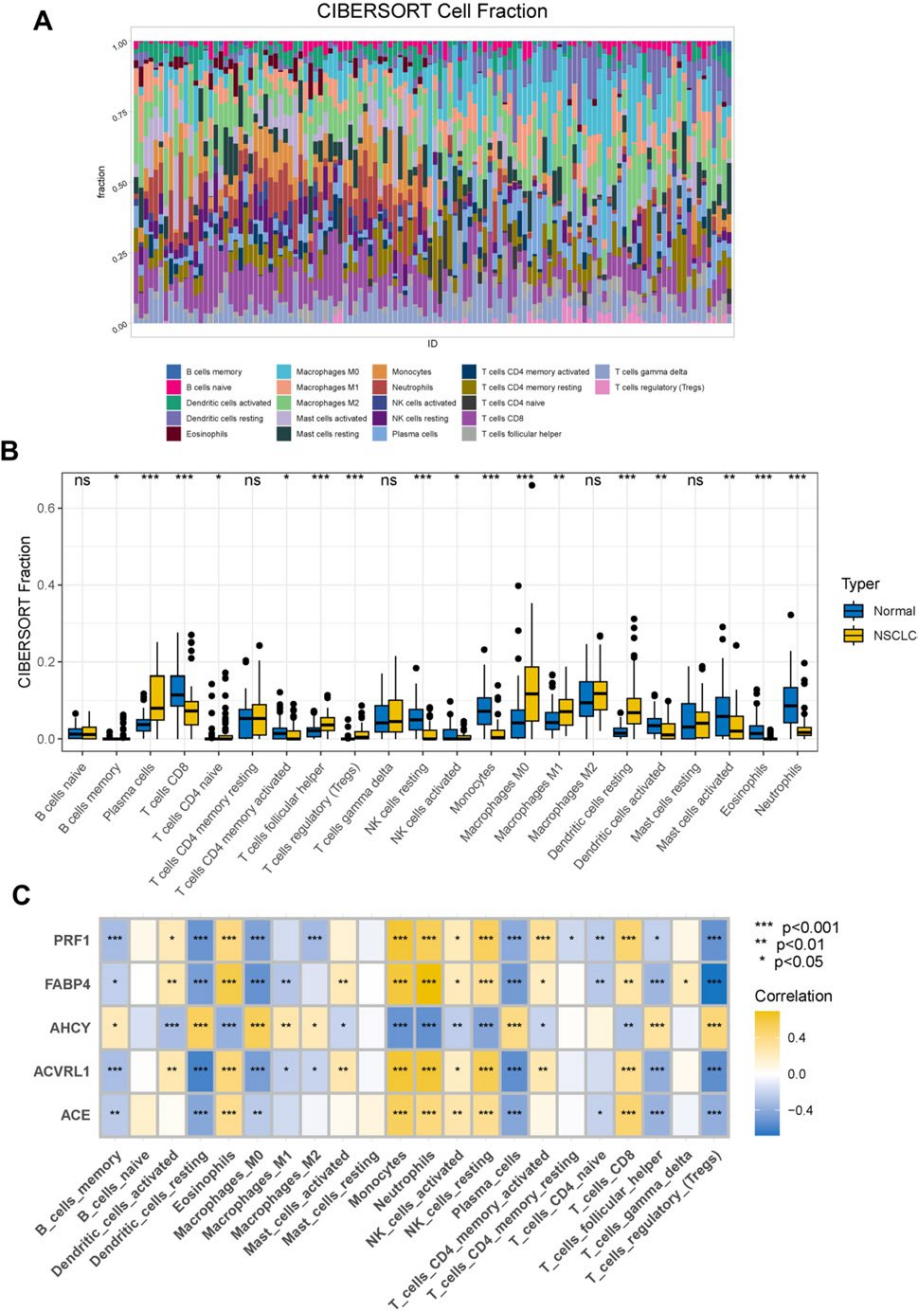
Immune infiltration landscape. **A** the stacked bar chart illustrates the relative proportions of immune cells in the first 60 NSCLC samples compared to the subsequent 60 normal lung tissue samples, **B** box plots for differential analysis of immune cells in two groups of samples, **C** heat map of the correlation between five core targets and immune cells

As illustrated in Fig. 6A and B, immune cells such as Plasma cells, T cells follicular helper, T cells regulatory (Tregs), Macrophages M0, and resting Dendritic cells were significantly positively correlated in NSCLC samples. In contrast, T cells CD8, resting NK cells, Monocytes, Eosinophils, and Neutrophils showed significant negative correlations. Furthermore, we examined the relationship between the expression levels of core targets and the immune microenvironment. Tregs, T cells follicular helper, and Macrophages M0 were significantly negatively correlated with ACVRL1, ACE, FABP4, and PRF1, but positively correlated with AHCY. Conversely, Neutrophils and resting NK cells exhibited significant positive correlations with ACVRL1, ACE, FABP4, and PRF1, while showing significant negative correlations with AHCY (Fig. 6C). These findings suggest that alterations in the immune microenvironment of NSCLC patients may be closely associated with the five candidate core targets.

### Prediction of affinity of BYJHD core components to candidate targets

**Fig. 7:**
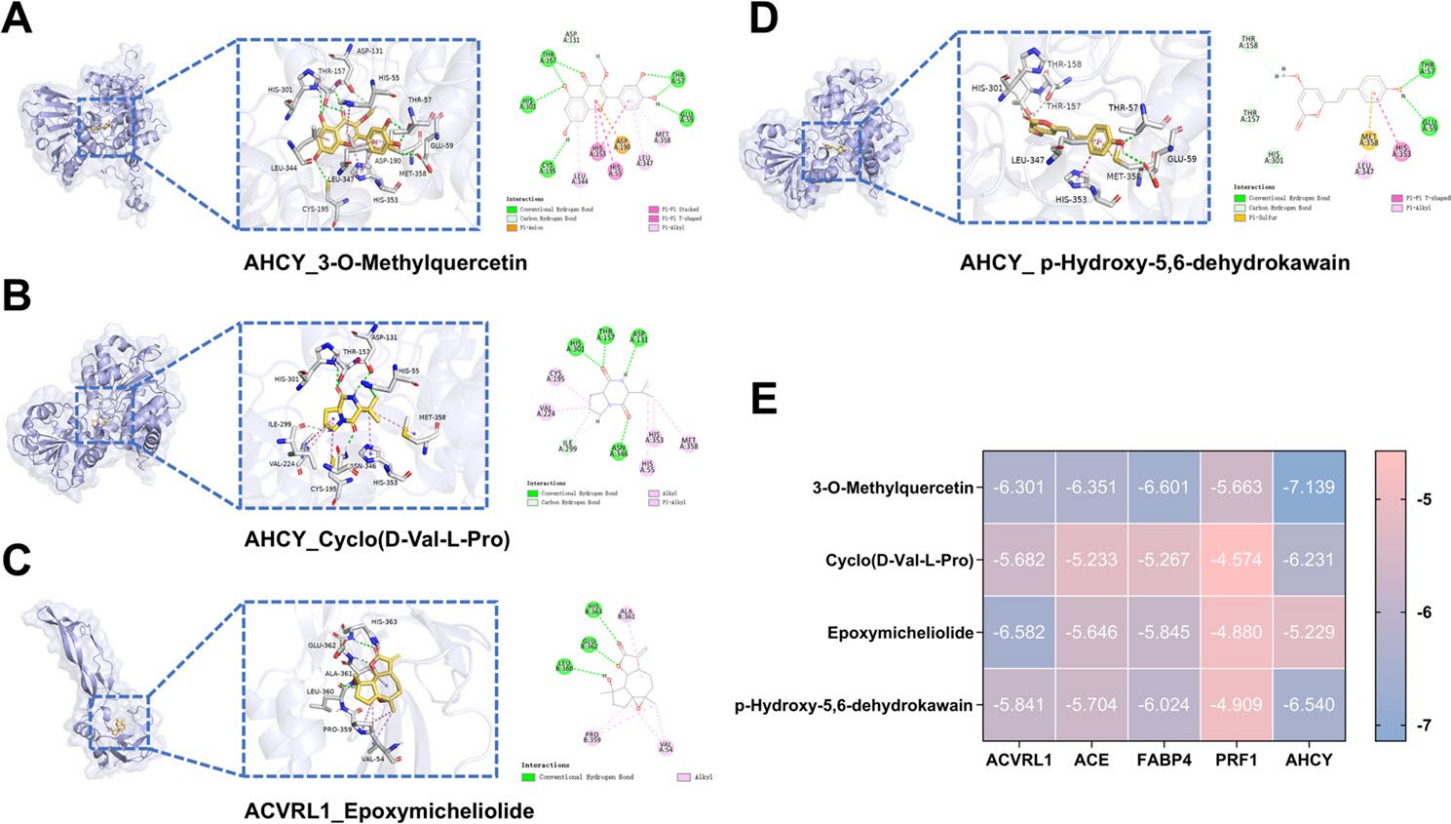
Molecular docking validates interaction of key components with core candidate targets. **A**–**D** Visualization of docking results and interaction patterns of key compounds in complex with target proteins. **E** Heat map of molecular docking binding energy

Molecular docking was performed to predict the interactions between the top four active compounds in the‘active component-target’network, ranked by degree (p-Hydroxy-5,6-dehydrokawain, 3-O-Methylquercetin, Cyclo (D-Val-L-Pro), and Epoxymicheliolide), and five candidate targets. Except for PRF1, the binding energies of the compounds with the other three targets were all below − 5 kcal/mol. Among these, 3-O-Methylquercetin exhibited the strongest binding affinity with AHCY, with a binding energy of -7.1392 kcal/mol. Cyclo (D-Val-L-Pro) demonstrated a binding energy of -6.2307 kcal/mol with AHCY, Epoxymicheliolide showed a binding energy of -6.5819 kcal/mol with ACVRL1, and p-Hydroxy-5,6-dehydrokawain exhibited a binding energy of -6.5404 kcal/mol with AHCY. These findings indicate strong interactions between the compounds and their respective targets. The interactions involved include the formation of hydrogen bonds, hydrophobic interactions, cation-π interactions, and π-π stacking forces, as shown in Fig. 7. Given the importance of PRF1 within the target network, it was retained and subjected to subsequent in vitro testing alongside other core targets.

### Effect of BYJHD on cell viability and cell migration of lung cancer cells

**Fig. 8:**
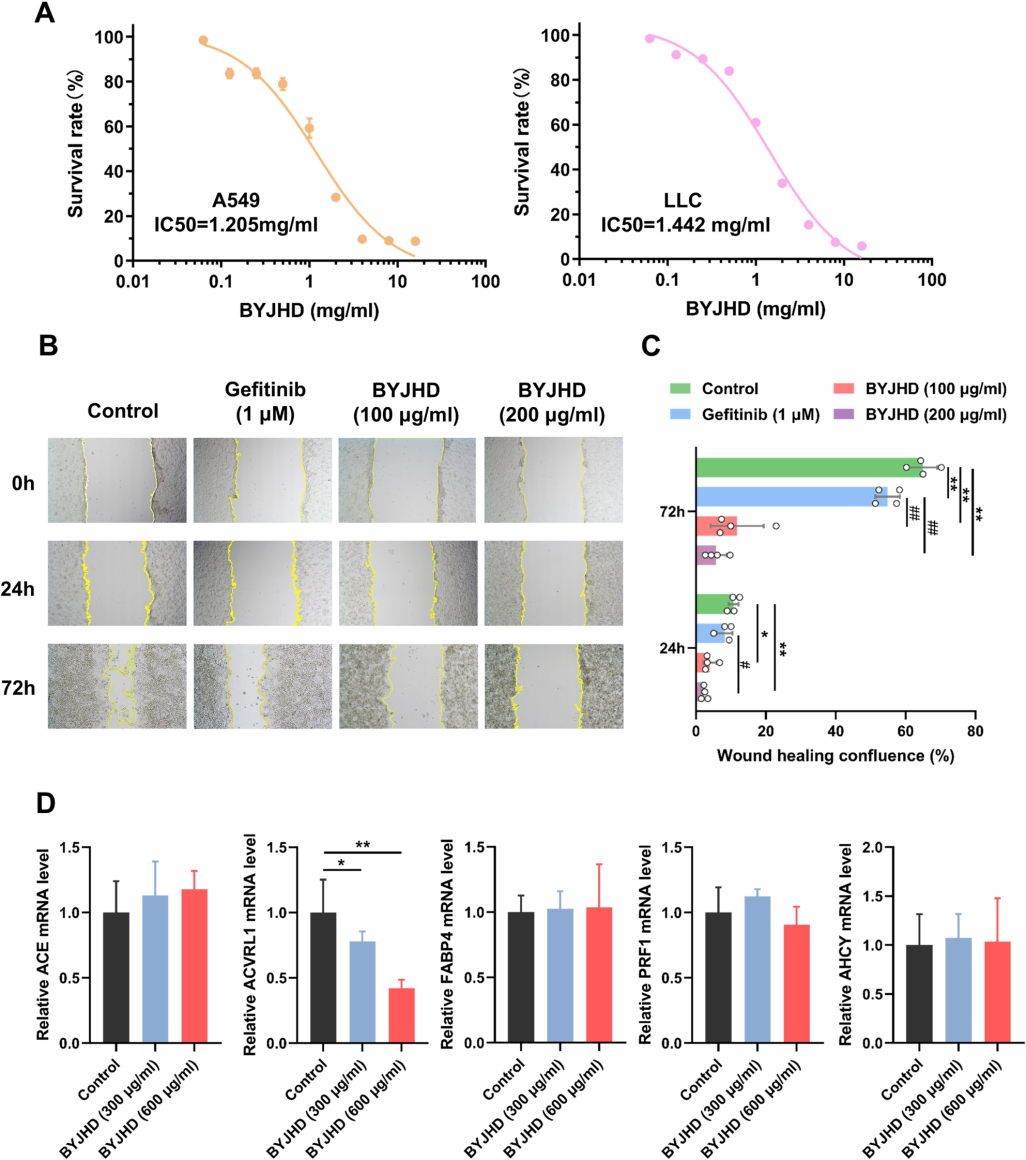
BYJHD reduces survival rate in A546 and LLC cell lines while inhibiting migration of A549 cells. **A** The viability of A549 and LLC cells was significantly reduced following treatment with various concentrations of BYJHD (0.0625, 0.125, 0.25, 0.5, 1, 2, 4, 8, and 16 mg/mL), **B**, **C** the effects of 1 µM gefitinib and BYJHD at concentrations of 100 and 200 µg/mL on A549 cell migration were assessed through scratch-healing assays at 24 h and 72 h. BYJHD effectively inhibited A549 cell migration, as indicated by impaired scratch closure. (D) mRNA expression levels of candidate targets (ACE, ACVRL1, FABP4, RPF1 and AHCY) in A549 cells after BYJHD treatment. All data are presented as the mean ± SD (*n* = 4). ^*^P < 0.05, ^**^P < 0.01, compared with the control group; ^#^*P* < 0.05, ^##^*P* < 0.05, compared with the gefitinib group

We evaluated the effects of varying concentrations of BYJHD on the viability of A549 and Lewis Lung Carcinoma cells (LLC) using the CCK-8 assay. Treatment with BYJHD at a concentration of 1 mg/mL resulted in a significant reduction in cell viability (below 70%) in both cell lines. The cytotoxic effects of BYJHD were dose-dependent, with IC50 values calculated as 1.205 mg/mL for A549 cells and 1.442 mg/mL for LLC cells, respectively (Fig. 8A). Furthermore, results from the scratch assay demonstrated that sub-toxic concentrations of BYJHD (100 and 200 µg/mL) markedly suppressed the migration of A549 non-small cell lung cancer cells, showing a significantly stronger inhibitory effect compared to 1 µM gefitinib at 72 h (Fig. 8B and C).

### Validation of candidate core targets at the mRNA expression level

To further validate the reliability of the core targets of BYJHD for NSCLC treatment screened by network pharmacology combined with machine learning, mRNA of A549 cells was quantified in vitro after treatment with different doses of BYJHD. The results showed that only ACVRL1 produced drug response with a significant decrease in mRNA expression level (Fig. 8D), predicting that ACVRL1 plays a key role in BYJHD treatment of NSCLC. No significant differences were observed at the mRNA level for the remaining targets, which may be related to their cell type and tissue-specific expression patterns.

### BYJHD inhibits tumour cell proliferation in vivo

**Fig. 9:**
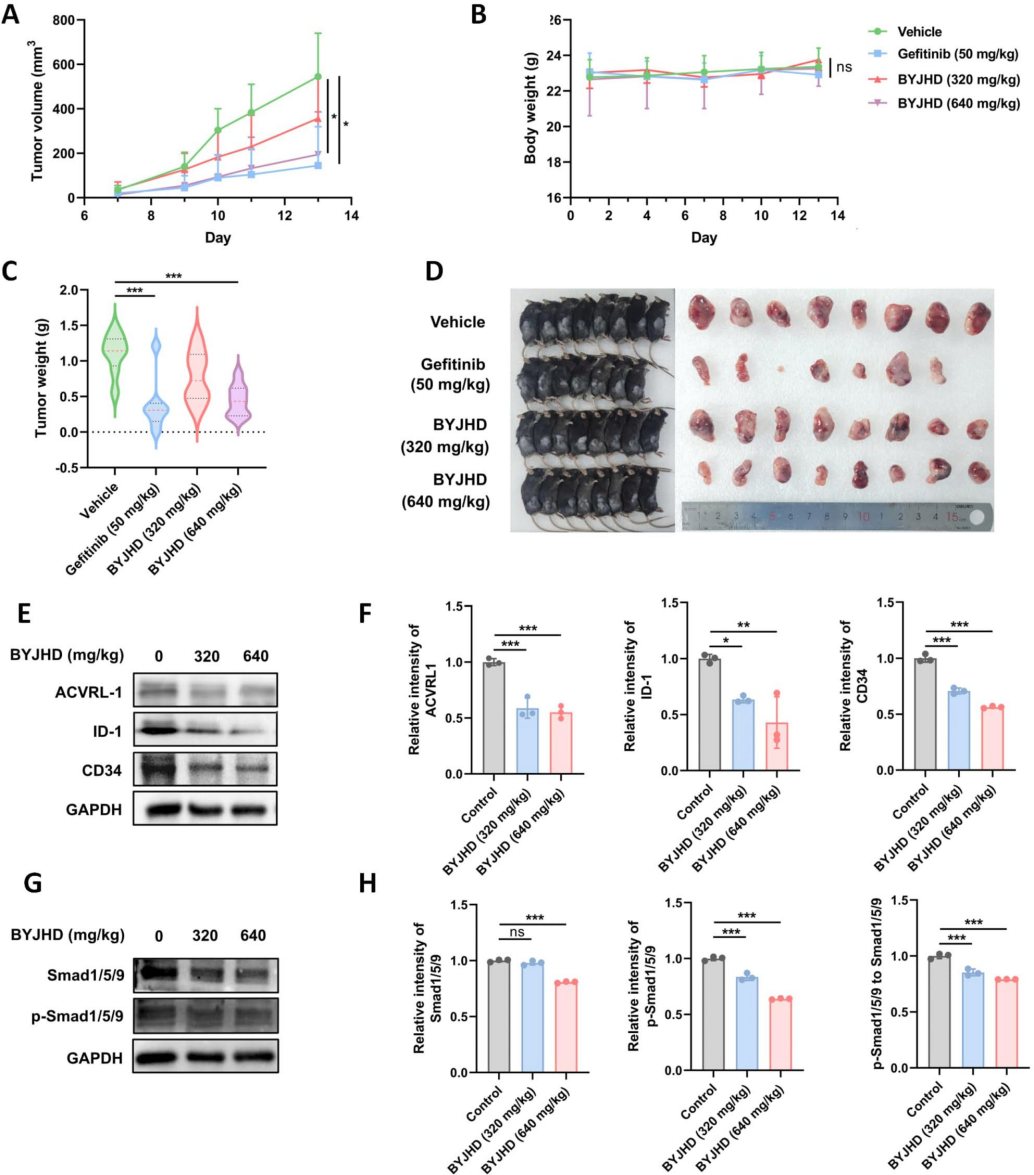
BYJHD inhibited tumor growth in Lewis lung carcinoma cell xenograft model. The mice were randomly divided into excipient group, gefitinib group, low/medium dose BYJHD group (320 mg/kg), and high dose group BYJHD group (640 mg/kg). **A** Changes in body weight of mice in each group during the treatment cycle, **B** tumour volumes for each group calculated from regular measurements of tumour size by calipers, **C** comparison of tumour weights after stripping the tumours from each group of mice. (D) Visual observation of mice and excised tumours in each group of mice, **E**, **F** Protein expression and quantitative analysis of ACVRL-1, ID-1, and CD34, **G**, **H** quantitative analysis of the expression levels of Smad1/5/9, p-Smad1/5/9 and the ratio between them. Data are presented as mean ± SD (*n* ≥ 7). *ns* represents no significance, ^*^P < 0.05, ^**^P < 0.01, ^***^P < 0.001, compared with the control group

The in vivo anti-tumour effect of BYJHD was evaluated in subcutaneous xenograft model mice. During the 14-day treatment period, the body weights of the mice all changed steadily, with no significant differences between the groups (Fig. 9A). The tumour volume increased accordingly with the continuous increase of time, but the tumour growth rate was significantly slower in the gefitinib group and the BYJHD high-dose group than in the vehicle group (Fig. 9B). In addition, the tumour weight of mice showed a trend of decreasing after BYJHD treatment, and the tumour-suppressing effect of BYJHD in the high-dose group was comparable to that of gefitinib (Fig. 9C and D).

### BYJHD exerts anti-tumor effects by inhibiting the activation of the ACVRL-1/Smad/ID-1 signaling pathway and suppressing CD34 expression

Based on the results of in vitro detection of mRNA expression of the core target, we focused on ACVRL-1 and its related proteins. Meanwhile, we identified GO- and KEGG-enriched signaling pathways associated with cancer-related transcriptional dysregulation, as well as biological processes involving endothelial cell proliferation, which are closely linked to the function of ACVRL-1 and its downstream signaling molecules. To further explore this, we analyzed the expression of ACVRL-1, the phosphorylation levels of Smad1/5/9, and the expression of ID-1 in tumor tissues. The results demonstrated that BYJHD treatment significantly downregulated the expression of ACVRL-1 and ID-1 while inhibiting the phosphorylation of Smad1/5/9 (p-Smad1/5/9 relative to total Smad1/5/9). Furthermore, treatment with 600 µg/mL BYJHD also reduced the total expression levels of Smad1/5/9. In addition, BYJHD markedly suppressed the expression of CD34 in tumor tissues. (Fig. 9E-H)

## Discussion

The latest report from the International Agency for Research on Cancer (IARC) on the global burden of cancer in 2022 revealed that lung cancer has overtaken breast cancer to once again become the most prevalent cancer worldwide (Bray et al. 2024). As the predominant form of lung cancer, NSCLC has benefited from progress in targeted therapy and immunotherapy. Nevertheless, the persistence of drug resistance, toxic side effects, and substantial treatment costs underscores the urgent demand for novel therapeutic approaches (Riely et al. 2024; Meyer mL, Fitzgerald et al. 2024). One key advantage of traditional Chinese medicine (TCM) lies in its use of multiple active components, which can exhibit synergistic effects by acting on multiple targets simultaneously (Ji et al. 2023). This aligns with TCM’s holistic perspective, which has gained increasing recognition with the emergence of interdisciplinary fields such as bioinformatics, systems biology, and computational biology. These disciplines have driven a paradigm shift in modern medicine from a reductionist to a holistic approach. Correspondingly, research strategies for disease diagnosis and treatment have transitioned from a focus on “single disease, single target, and single drug” to “multi-target and systematic regulation,” emphasizing the importance of analyzing complex diseases from a systems biology perspective (Greene and Loscalzo 2017; Nogales et al. 2022). We therefore used network pharmacology and machine learning approaches combined with experimental validation to elucidate the potential therapeutic mechanisms of BYJHD in NSCLC. To our knowledge, this is the first study to systematically investigate the material basis and potential mechanisms underlying BYJHD’s antitumor activity through an integrated approach.

Synthesizing the results of metabolomics, bioinformatics and network pharmacology studies, 20 compounds such as p-Hydroxy-5,6-dehydrokawain, 3-O-Methylquercetin, Cyclo(D-Val-L-Pro), Epoxymicheliolide, Medicarpin, rosmarinic acid, and 20 other compounds were identified as the core pharmacodynamic components of BYJHD exerting anti-NSCLC. Recently, Zhou et al. reported that Epoxymicheliolide reduced radiation-induced cellular senescence and extracellular matrix formation by interfering with NF-κB and TGF-β/SMAD pathways in lung cancer mice (Zhou et al. 2024). Pintha et al. found an inhibitory effect of rosmarinic acid on oxidative stress, inflammation and metastatic capacity of PM-exposed A549 cells through C-Jun, P-65-NF-Κb and Akt signalling pathways (Pintha et al. 2021). Rosmarinic acid has also been reported to reverse cisplatin resistance in non-small cell lung cancer by activating the MAPK signalling pathway (Liao et al. 2020). Among them, Nobiletin was reported to enhance chemosensitivity to adriamycin by modulating the Akt/GSK3β/β-Catenin/MYCN/MRP1 signalling pathway in A549 human non-small cell lung cancer cells (Moon et al. 2018). Meanwhile, Nobiletin resist immune evasion by modulating miR-197/STAT3/PD-L1 signalling in non-small cell lung cancer (NSCLC) cells (Sp et al. 2021). Combined with these findings, it also validates the reliability of our identification of active natural products against NSCLC in BYJHD, in which drugs that have not been reported to have antitumour activity (e.g. p-Hydroxy-5,6-dehydrokawain, Cyclo(D-Val-L-Pro), Tetramethylcurcumin, Epoxymicheliolide, etc.) It is valuable for further research.

We constructed PPI networks from 54 potential targets identified through data mining and analyzed their functions by GO and KEGG enrichment. A total of 35 signalling pathways (q value < 0.05) were ultimately identified for BYJHD intervention in NSCLC, with most cancer-related pathways emphasizing on effects on the tumour microenvironment and vascular-related factors. Since complex networks analyses often produce false-positive results, and because machine learning approaches are increasingly used to identify core targets is becoming a trend (Noor et al. 2023; Xu et al. 2021), we applied three machine learning models to more precisely determine the effective targets of BYJHD for NSCLC treatment. These models identified five candidate core targets (ACVRL1, ACE, FABP4, PRF1, and AHCY) from the 54 potential targets.

In *vitro*, BYJHD exhibited cytotoxic effects and inhibited the growth of lung cancer cells. An entity screen in non-small cell lung cancer cells A549 identified the target gene ACVRL1 that produces expression changes in response to drug treatment. ACVRL1 (Activin A Receptor-Like Type 1), also known as ALK1, is a type I transmembrane serine/threonine kinase receptor of the transforming growth factor-beta (TGF-β) superfamily, which is predominantly expressed in vascular endothelial cells, and has an important role in the regulation of angiogenesis and endothelial homeostasis (Schoonderwoerd et al. 2020). Neumorous studies have reported elevated ACVRL1 expression in certain tumours such as hepatocellular carcinoma, breast cancer (Cunha et al. 2015), where it is associated with aberrant angiogenesis and tumour progression (Cunha and Pietras 2011; Hawinkels et al. 2013). Anti-tumour strategies targeting ACVRL1 are also gradually attracting academic attention. Inhibition of ACVRL1 expression in transgenic tumour-bearing mice or treatment with ALK1-Fc fusion protein RAP-041 inhibits angiogenesis to delay tumour growth and progression (Cunha et al. 2010). In clinical studies, the ligand trap (dalantercept) (Jimeno et al. 2016) and the monoclonal antibody PF-03446962 of ACVRL1 have demonstrated desirable anti-tumour efficacy (Goff et al. 2016; Simonelli et al. 2016). Notably, a study showed that Hsa_circ_0129047 regulates the miR-375/ACVRL1 axis to slow the progression of lung adenocarcinoma (Fan et al. 2022). In this study, BYJHD inhibited the transcriptional level of ACVRL1 in the A549 cell line and the expression of ACVRL1 protein in tumour-bearing mice.

Binding of ACVRL1 to ligands initiates the downstream Smad signalling pathway, which can mediate signalling in endothelial cells through phosphorylation of SMAD1/5/8 ^30^. Smads protein activation after nuclear translocation regulates the transcription of specific target genes such as (ID-1) to promote endothelial cell proliferation (Hawinkels et al. 2013). ACVRL1 blockade in HUVEC inhibits downstream Smad1/5/8 phosphorylation and ID-1 expression (Hu-Lowe et al. 2011), Downregulation of Smad1/5/8 phosphorylation levels as well as ID-1 expression was also observed in our study. On the other hand, CD34 is often used to assess the microvessel density (MVD) of tumour tissues, including NSCLC, and is closely related to tumour angiogenesis, invasiveness, and microenvironmental constitutive cellular activity (Tanaka et al. 2001, 2003; Schulze et al. 2020). The expression of CD34 in tumour tissues of BYJHD-treated mice was also significantly reduced. In summary, we speculate that BYJHD exerts its anti-NSCLC activity through the ACVRL-1/Smad/ID-1 axis and the inhibition of CD expression, thereby regulating the tumour microenvironment and inhibiting tumour angiogenesis. Future work should include genetic or pharmacological inhibition of ACVRL1 to determine whether its suppression alone recapitulates the anti-tumor and anti-angiogenic effects of BYJHD, thereby confirming its indispensable role in mediating therapeutic outcomes.

In conclusion, starting from the knowledge of BYJHD in the treatment of NSCLC from the theoretical system of Chinese medicine and the experience of clinical treatment, we used the strategies of computational biology and systems biology to validate the anti-NSCLC activity of BYJHD in the laboratory and attempted to excavate the relevant molecular mechanisms. Admittedly, our study has some limitations. As the integration and analysis of data based on existing knowledge is limited by databases and algorithms, the results of the study may not be exactly the same as the actual structure. While the identification of core targets based on target contribution improves the accuracy of target identification, it sacrifices the possibility of other potential pharmacodynamic targets being identified. Systematic research is based on experimental design and progresses step by step based on the results of each stage, which may lead to more research findings being overlooked. In addition, more work is still needed to study the mechanism of active ingredient monomers and to investigate the drug-drug interactions between different monomers.

## Conclusion

In this study, we successfully identified the material basis and molecular biological mechanism of BYJHD for the treatment of NSCLC through metabolomics, network pharmacology, machine learning, molecular docking, and in vivo and in vitro experimental validation. BYJHD can down-regulate the phosphorylation of Smad protein by inhibiting the expression of tumour ACVRL1, and reduce the expression of the target gene ID-1 and the surface marker CD34 in the tumour microenvironment to regulate tumour angiogenesis, leading to anti-NSCLC effects. Although further experimental studies are needed to confirm additional mechanisms, this work systematically explores potential targets and signalling pathways related to the anti-NSCLC activity of BYJHD, which provides a methodology and reference for future pharmacological studies.

## Electronic Supplementary Material

Below is the link to the electronic supplementary material.

Supplementary Material 1

## Data Availability

All data supporting the findings of this study are available within the article or from the corresponding author on reasonable request.
