# Supplementary material for: Anti-angiogenesis effect of Baiying Juhua Decoction on the non-small cell lung cancer: integrating pharmacology, multi-machine learning and experimental investigation
Source: Bioresour Bioprocess. 2026 Jan 24;13(1):5. doi: 10.1186/s40643-025-00993-3 (PMC12831725; doi:10.1186/s40643-025-00993-3)
Supplement: Supplementary file 1 — Supplementary Material 1 [file 40643_2025_993_MOESM1_ESM.docx]

**Supporting Information**

**Anti-angiogenesis effect of Baiying Juhua decoction on the non-small cell lung cancer: integrating pharmacology, multi-machine learning and experimental investigation**

Xiangwei Meng^a, b^, Yuan Cao^b^, Qi Shen^b^, Hongyu Zhu^c,^ *, Jianqiao Zhang^b, d,^ *, Mingxin Dong^a,^ *

^a^ Department of Medicinal Chemistry, School of Pharmacy, Qingdao University, Qingdao, 266021, China.

^b^ Department of Drug Clinical Trials, Zibo Central Hospital Affiliated to Binzhou Medical University, Zibo, 255000, China.

^c^ Central Laboratory, The Affiliated Xuzhou Municipal Hospital of Xuzhou Medical University, Xuzhou, 221000, China.

^d^ School of Pharmacy, Key Laboratory of Molecular Pharmacology and Drug Evaluation (Yantai University), Ministry of Education, Collaborative Innovation Center of Advanced Drug Delivery System and Biotech Drugs in Universities of Shandong, Yantai University, Yantai, 264005, China.

***Corresponding author**: Hongyu Zhu, E-mail: neuron.hy.zhu@outlook.com. Jianqiao Zhang, E-mail: jaybridge@163.com. Mingxin Dong, E-mail: mxdong64@qdu.edu.cn.

**Supplementary figures**

Table S1. Information on the top 20 core compounds ranked by degree.

| Name | Degree | BC | CC |
| --- | --- | --- | --- |
| p-Hydroxy-5,6-dehydrokawain | 12 | 0.026528544 | 0.381578947 |
| 3-O-Methylquercetin | 10 | 0.019586591 | 0.375 |
| Cyclo(D-Val-L-Pro) | 9 | 0.013992399 | 0.3625 |
| Epoxymicheliolide | 8 | 0.008335438 | 0.353658537 |
| Medicarpin | 8 | 0.008915025 | 0.356557377 |
| rosmarinic acid | 7 | 0.006067354 | 0.350806452 |
| Tetramethylcurcumin | 7 | 0.006288488 | 0.33984375 |
| trans-5-Hydroxyferulic acid | 6 | 0.005440662 | 0.353658537 |
| Citropten | 5 | 0.005741598 | 0.33984375 |
| Monodictyxanthone | 5 | 0.003069668 | 0.332061069 |
| Skimmin | 5 | 0.00556861 | 0.348 |
| 2',5,6',7-Tetraacetoxyflavanone | 4 | 0.003163613 | 0.342519685 |
| 6-Dehydropetaso | 4 | 0.002767364 | 0.33984375 |
| Ethyl salicylate | 4 | 0.001060342 | 0.315217391 |
| Hexylitaconic acid | 4 | 0.00154628 | 0.319852941 |
| trans-Fertaric acid | 4 | 0.001433566 | 0.332061069 |
| Flavinantine | 3 | 0.001271724 | 0.322222222 |
| Nitogenin | 3 | 6.35E-04 | 0.310714286 |
| Nobiletin | 3 | 0.001253241 | 0.327067669 |
| Syringic acid | 3 | 9.56E-04 | 0.329545455 |

BC: BetweennessCentrality, CC: ClosenessCentrality

Table S2. Information on the top 20 core targets ranked by degree.

| Gene | Degree | BC | CC |
| --- | --- | --- | --- |
| PPARG | 44 | 0.080602 | 0.854839 |
| MMP9 | 43 | 0.061266 | 0.84127 |
| KDR | 37 | 0.036448 | 0.768116 |
| ACE | 36 | 0.029776 | 0.757143 |
| DPP4 | 34 | 0.044394 | 0.736111 |
| KIT | 33 | 0.026232 | 0.726027 |
| ABCB1 | 32 | 0.032898 | 0.716216 |
| MMP1 | 30 | 0.015687 | 0.697368 |
| SELP | 29 | 0.012307 | 0.688312 |
| GZMB | 28 | 0.013892 | 0.679487 |
| ADRB2 | 28 | 0.017209 | 0.670886 |
| FABP4 | 28 | 0.025895 | 0.679487 |
| BCHE | 27 | 0.032911 | 0.670886 |
| F3 | 26 | 0.006629 | 0.6625 |
| FYN | 26 | 0.019276 | 0.6625 |
| TEK | 26 | 0.010124 | 0.6625 |
| EDNRB | 25 | 0.009477 | 0.646341 |
| MME | 25 | 0.008792 | 0.654321 |
| HDAC1 | 24 | 0.032386 | 0.646341 |
| PRF1 | 24 | 0.007989 | 0.646341 |

BC: Betweenness Centrality, CC: Closeness Centrality
